# A Case of Hernia-Cerebri, or Congenital Encephalocele

**Published:** 1859-09

**Authors:** W. S. Forwood

**Affiliations:** Darlington, Md.


*A Case of Hernia-Cerebri, or Congenital Encephalocele.* By W. S. Forwood, M. D., of Darlington, Md.

January 31, 1859. This morning I was called to see an infant that had just been born, with the expectation on the part of the parents that medical or surgical aid could be rendered available in reducing or changing an abnormal development. The facts are as follows:

P. M., a healthy colored woman, about 36 years of age, having previously given birth to six or seven healthy, well-formed children, was delivered of twins, by a midwife, immediately before I was sent for. One of them, the male, was of good size, and well developed in all respects; the other, a female, was near one-third smaller, and presented a large, elongated, pyriform tumor upon the back part of the head; occupying the region of the *os occipitis.*

This tumor and the child's head both resembled very closely in shape, though not so large, the monster fœtus that some years ago came under the observation of Dr. Rohrer, of Philadelphia, and is figured by Prof. Meigs, in his work on *Obstetrics,* (1st ed. pp. 190, 375.) The head of the infant was quite small, and the tumor was as large as the head, or about 2½ by 4 inches in its diameter. This tumor, however, was not of a hydrocephalic character, as was Dr. Rohrer's case. It contained, principally solid matter, as if the brain had protruded from the cavity of the cranium, (which was undoubtedly the case,) together with a small quantity of liquid. No bone could be felt beneath the cranial attachment of the tumor, (which covered a space of about an inch and a quarter in diameter;) and this absence of a partition between the pendulous attachment and the encephalon, clearly established the case as one of *hernia-cerebri,* without the conclusive testimony of dissection. The head and features were hideously deformed and distorted. There was no elevation of the head above the eyes; the cranium retreated directly back from that point; the eyes were protuberant; the chin receding; and the facial integuments wrinkled and shrunken. The pedicle of the tumor was supplied with hair, like the scalp, as in the case above referred to; the remainder was covered with ordinary skin, appearing like that upon the neck. The neck was so short that it could scarcely be said to have any; the head seemed to be united immediately to the shoulders. The tumor was not so large as to offer any impediment to labor.

This infant lived nineteen days, although it could not, from physcal incapacity, draw any milk from its mother's breast, and could with great difficulty swallow when fluids were put into its mouth.— It never cried, but would occasionally make a slight groaning noise deep in the throat. Its eyes were kept almost constantly closed; and during the greater part of its life it was affected with weak and transient spasms. Of course nothing was attempted towards changing the state of things; and the family were informed that, from the nature of the case, it was impossible for the child to live more than a few days, or, at most, a few weeks. Unfortunately no post-mortem examination was had, as the child was buried some days before I was informed of its death.

In my comparatively limited range of reading, I have not met with a report of any case similar to this, except that of Dr. Rohrer's, and his differed from the present one, by being a case of hydrocephalus externus; the water having formed in such a large quantity as to make it necessary for it to seek an outlet from the cavity of the cranium.

The case reported by Mr. J. Z. Laurence, in the *London Lancet* for Dec., (American re-print,) under the head of encephalocele, it would seem by the history and the illustration accompanying it, was improperly so called—the enormous tumor being composed, like Dr. Rohrer's case, altogether of fluid, with the exception of a small solid mass at the base of the pedicle of the sac, and there being no consequent deformity of the head.

Since the above was penned, I have read the report of another case of encephalocele, by Mr. J. B. Thompson, in the August No. of the *Lancet,* (re-print,) for the present year, (1859.) This case is very similar to that of Mr. Laurence; the sac was filled with water, and post-mortem examination revealed the presence of a portion of the cerebellum, about the size of a walnut, protruding beyond the cranial walls. He does not allude to the existence of any deformity of the head.

The interesting features of the case that I have noticed are, the large amount of brain extended, as indicated by the feel of the tumor, and by the evident deficiency within the diminished and deformed cranium, and the fact of the child surviving so long after birth in this condition, without taking scarcely any nourishment.

Mr. Laurence states that only about eighty cases of encephalocele (or *hydro-cephalocele* as some of the cases might more properly be called,) have been recorded in medical history.

While upon the subject of infantile cephalic tumors, it may not be out of place to add a short report of another variety. Some years ago, while a pupil of Dr. Warrington's in the *Philadelphia Obstetric Institute*, I met with a case in which a tumor formed upon the head of a child after its birth; at least it was not observed until the following day, when my attention was drawn to it by the nurse, who had just discovered it. It occupied (as I see by referring to my notes taken at the time) the upper portion of the leg of the lambdoidal suture, and was about the size of an almond when first noticed. It was rather soft, but not at all fluctuating or painful to the touch. The infant was full sized, and in all other respects seemed perfectly well. This tumor continued to grow for two or three weeks, having in that time spread over a surface of about three inches in diameter, and become elevated rather more than half an inch.

Dr. Warrington visited the child with me on one or two occasions, and considered it as a very singular case. He was inclined to the opinion at first that it was a case of sanguineous effusion in the cellular tissue of the scalp, but afterwards doubted this view. As the child did not appear to suffer at all, we resorted to no treatment except the constant application of slippery elm poultices. At about the third week, when the tumor had attained the above mentioned size it ceased to enlarge, but maintained this size without diminishing in the least for two weeks longer, when the term of my pupilage having expired, I left the city, and have never heard anything of the case since.

These crude and fragmentary contributions to pathology, though possessing little value in themselves, may, when added to the general stock, be found as usefnl mites.—*Phil. Med. Peporter.*

